# Comparison of oncological and functional outcomes in Lower-limb osteosarcoma pediatric patients: a large single-center retrospective cohort study

**DOI:** 10.1097/JS9.0000000000001340

**Published:** 2024-03-12

**Authors:** Mingxian Xu, Jian Tu, Yongfu Huang, Bo Wang, Zhiqiang Zhao, Tiao Lin, Gang Huang, Junqiang Yin, Xianbiao Xie, Jingnan Shen, Changye Zou

**Affiliations:** a Department of Musculoskeletal Oncology, The First Affiliated Hospital, Sun Yat-sen University; b Guangdong Provincial Key Laboratory of Orthopedics and Traumatology, The First Affiliated Hospital, Sun Yat-sen University, Guangzhou, China

**Keywords:** limb salvage, lower limb, osteosarcoma, outcomes, pediatric

## Abstract

**Objective:**

Treating pediatric osteosarcoma in long bones is challenging due to skeletal immaturity, which restricts the generalizability of insights derived from adult patients. Are there disparities in outcomes? How should surgical protocols be tailored for children of varying ages? What are the specific postoperative complications? A large single-center retrospective cohort study of 345 patients under 14 years old with lower-limb osteosarcoma treated in our department since 2000 was conducted to address these inquiries.

**Methods:**

A retrospective analysis of 345 pediatric patients with lower-limb osteosarcoma admitted to our department between 2000 and 2019 was conducted. Clinical and functional outcomes were compared based on age groups, surgical methods, type of prosthesis, and primary tumor location. Patients were divided into the low-age group (≤10 y old) and the high-age group (>10 y old). Overall survival rate (OS), progressionfree survival rate (PFS), and prosthesis survival rate were assessed using Kaplan–Meier curves, nonparametric survival analysis (log-rank test), and Univariate cox regression were used for comparison. The incidence of complications, local relapse rate (LRR), metastasis rate, final limb-salvage, and amputation rate, and Musculoskeletal Tumor Society (MSTS) score of different independent groups were further evaluated using *χ*^2^ test or Fisher’s exact test, and *t*-test was employed to evaluate the measurement data.

**Results:**

The average age of the patients was 11.10±2.32 years ranging from 4 to 14 y, with an average follow-up duration of 48.17 months. The 5, 10, and 15-year OS rates were 50.3%, 43.8%, and 37.9%, respectively. The progression-free survival rate was 44.8% at 5 years and 41.1% at 10 years. The final limb salvage rate was 61.45%, while the final amputation rate was 38.55%. The low-age group had a higher amputation rate compared with the high-age group (48.00% vs. 33.18%, *P*=0.009). The overall LRR was 9.28%, and the incidence of metastasis was 28.99%. The LRR of the limb-salvage group was higher than the amputation group (*P*=0.004). The low-age group experienced more prosthesis-related complications than the high-age group (*P*=0.001). The most common prosthesis-related complication in the low-age group was soft-tissue failure, while the periprosthetic infection was most frequent in the high-age group. The high-age group had a higher cumulative prosthesis survival compared with the low-age group (*P*=0.0097). Modular prosthesis showed better MSTS scores and higher cumulative prosthetic survival than expandable prosthesis in pediatric patients (*P*<0.05).

**Conclusion:**

Limb preservation in pediatric patients becomes increasingly efficacious with advancing age, while consideration of amputation is warranted for younger patients. The prevailing postoperative complications associated with prosthesis encompass soft tissue failure and periprosthetic infection. Younger patients diagnosed with lower limb osteosarcoma exhibit a heightened amputation rate and a greater incidence of prosthesis-related complications.

## Introduction

Highlights

- Question: Treating pediatric osteosarcoma in long bones is challenging due to skeletal immaturity, which restricts the generalizability of insights derived from adult patients. Are there disparities in outcomes? How should surgical protocols be tailored for children of varying ages? What are the specific postoperative complications? What are the treatment outcomes and characteristics of lower limb osteosarcoma patients in pediatric under 14 years old?
- Findings: This retrospective analysis involved 345 patients under 14 years old with lower-limb osteosarcoma. The amputation rate is higher in younger patients, but limb preservation becomes more effective with increasing age. After limb salvage surgery in younger children, there are more prosthesis-related complications, particularly soft tissue failure, which differs from complications in adults.
- Meaning: Treatment of osteosarcoma in children lacks high-quality and large-scale research. This study provides the discipline with the largest known single-center detailed treatment outcomes and the characteristics of lower-limb osteosarcoma patients under 14 years old.

Osteosarcomas are quite rare with most cases typically diagnosed in adolescents or individuals over the age of 60, affecting around 5.2 children per million per year^1^. The peak incidence in adolescents is frequently attributed to the growth that occurs during puberty, with an earlier peak in girls than in boys^2^. The survival rate of individuals with detectable metastasis at other sites is less than 20%^2^. It most commonly occurs in the metaphyseal regions of the long bones, with around 61% of cases occurring in the lower limbs, especially the distal femur and proximal tibia^1,3^. Neoadjuvant chemotherapy and wide surgical resection have made limb salvage surgery possible in over 80% of osteosarcoma patients without compromising oncological outcomes^4^.

Limb salvage surgery is now the preferred treatment for malignant tumors of the extremities, particularly in pediatric patients. While more challenges may be faced when treating pediatric patients compared with adult patients due to bone immaturity and poor soft tissue coverage. Firstly, for very young patients, limb salvage is quite difficult because limb length discrepancy is inevitable even with an expandable prosthesis, thus amputation or limb salvage in low-age pediatric patients is the difficult choice. Secondly, how to reconstruct the limb after tumor removal^5^ and achieve optimal functional outcomes with minimal complications is challenging. Limb salvage with expandable or modular prosthesis has been the mainstream choice, while compared with skeletally mature patients, adolescent patients have different complications after endoprosthesis replacement, which may vary at different ages due to ongoing bone development in both circumference and length^6^. Thirdly, pediatric patients exhibit a longer survival cycle and higher daily activity level in contrast to adult patients, which may increase the incidence of prosthesis-related complications. Thus, pediatric osteosarcoma patients undergoing limb salvage surgery may encounter a higher incidence of complications and necessitate multiple surgical revisions in comparison to adult patients.

However, there is limited literature on pediatric osteosarcoma due to the rarity of the disease. Understanding the potential outcomes, risks, and benefits of each surgical option and endoprosthesis type is crucial for pediatric patients of different ages. This study aimed to investigate the specific characteristics of long-term oncological and functional outcomes in 345 children with osteosarcoma in the lower limb.

## Patients and methods

### Clinical data and information collection

A total of 345 pediatric patients under 14 years old with lower-limb osteosarcoma were included in this large single-center retrospective cohort study. The standard treatment for most of these patients typically involves complete surgical resection for those with localized disease and a neoadjuvant MAP regimen of high-dose methotrexate, doxorubicin, and cisplatin. Patients with nonresectable or widely metastatic osteosarcoma are generally treated with similar systemic therapy options, including MAP chemotherapy and local tumor control^2,7^. All patients received standard chemotherapy^8^ (One cycle of neoadjuvant chemotherapy including HDMTX-CF (10–12 g/m^2^), adriamycin (ADM, 50 mg/m^2^) + cisplatin (DDP, 100 mg/m^2^), and ifosfamide (IFO, 12.5 mg/m^2^) was prescribed for osteosarcoma) and tumor resection surgery in our hospital from January 2000 to May 2019. The general characteristics of patients and clinical data including tumor site, Enneking stage, oncological outcomes (local recurrence and distant metastases), operation and reconstruction procedures, survival status, complications, and revision surgery were recorded. The patients were followed-up as described before^8^. The function of the patients was assessed using the Musculoskeletal Society scoring system (MSTS)^9^. The research was conducted according to the principles of the World Medical Association Declaration of Helsinki. The research was retrospectively registered, and all subjects gave written informed consent. This retrospective study has been reported in line with the STROCSS criteria^10^. Supplemental Digital Content 1, http://links.lww.com/JS9/C151.

The overall survival rate (OS), progression-free survival rate (PFS), and cumulative prosthesis survival rate were assessed utilizing Kaplan–Meier curves and were compared using nonparametric survival analysis (Log-rank test) and Univariate cox regression. The differences in incidence of complications, local relapse rate (LRR) and metastasis rate, final limb-salvage and amputation rate, and MSTS score among independent groups were assessed the differences by using a *χ*^2^ test or Fisher’s exact test for enumeration data, and independent sample *t*-test for measurement data. A *P* value less than 0.05 was considered statistically significant.

**Figure 1:**
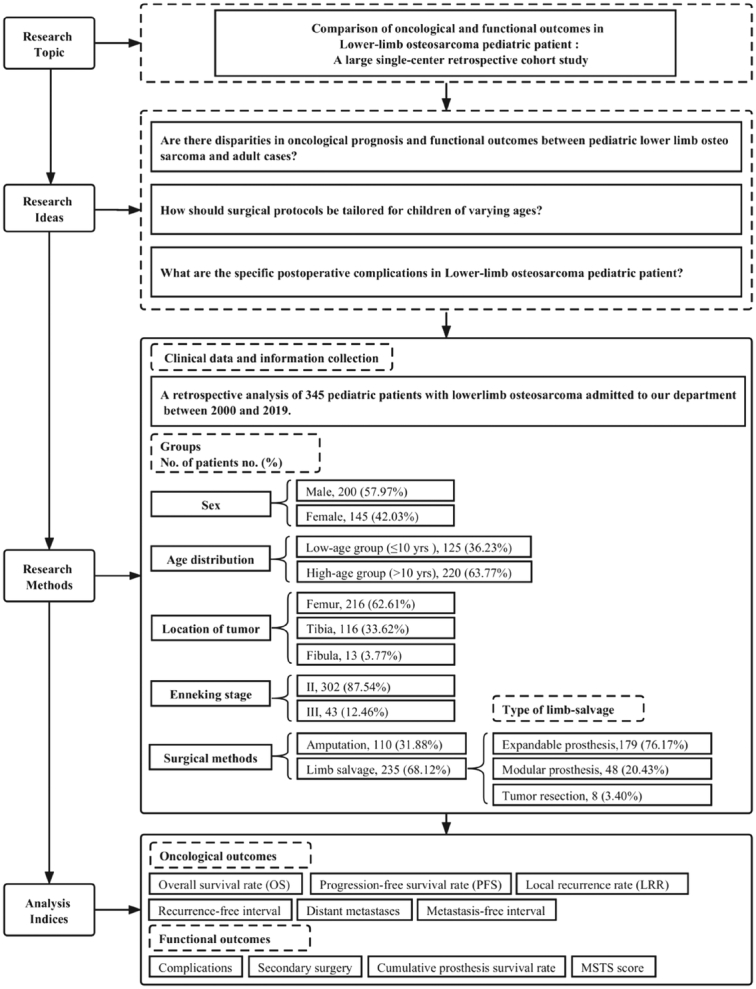
Schematic flow diagram of the study.

In order to compare the complications of different independent groups, we further uniformly classified the type of complications in limb salvage and amputation according to the Henderson ER’s^11^ failure mode classification for tumor endoprosthesis. The complications of limb salvage were classified into five categories: Type 1, soft tissue failure (insufficient soft tissue structural support) requiring further operation. Type 2, aseptic loosening requiring endoprosthesis revision. Type3, structural failure requires surgical intervention (periprosthetic fracture, implant fracture, bushing wear). Type4, periprosthetic infection (deep surgical site infection not amenable to implant retention). Type 5, tumor progression (local tumor relapse requiring surgical resection and/or revision). The complications of amputation were classified into three categories: Type A. Soft tissue failure (aseptic wound disruption and/or insufficient soft tissue structural support) requiring operation. Type B. Infection (superficial surgical site infection, deep surgical site infection, organ or space surgical site infection). Type C. Tumor progression (local tumor relapse requiring surgical resection). The schematic workflow of the study is presented in Figure 1.

## Results

### General data

From January 2000 to May 2019, a total of 345 pediatric patients (216 femurs, 116 tibias, 13 fibulas) with lower-limb osteosarcoma were included in this study. The average age was 11.10±2.32 years old (range 4 to 14 y old). Based on the characteristics of the included patients’ age distribution, we divided the age into the Low-age group (≤10 y old) and the High-age group (>10 y old). There were 125 patients were included in the low-age group (36.23%) and 220 patients were included in the high-age group (63.77%). Demographic, tumor stages, and surgical data are shown in Table 1. At the time of the study, 167 of the 345 patients were alive, 156 patients had succumbed to their disease, 22 patients had moved, and updated contact information was not available. The mean follow-up of patients was 48.17 months (range 1–243 months) (Table 1).

**Table 1:** Clinical features of including patients.

Variable	Femur	Tibia	Fibula	Total no. (%)
No.(%) of patients	216 (62.61)	116 (33.62)	13 (3.77)	345 (100)
No. Sex				
Male	124	68	8	200 (57.97)
Female	92	48	5	145 (42.03)
No. Age of diagnosis, y				
Mean (std)	10.92 (2.34)	11.53 (2.24)	10.92 (2.40)	11.10 (2.32)
Median (range)	11 (4-14)	12 (6-14)	11 (6-14)	12 (4-14)
No. (%) Age distribution				
≤10	83 (38.43)	37 (31.90)	5 (38.46)	125(36.23)
>10	133 (61.57)	79 (68.10)	8 (61.54)	220(63.77)
No. Location of tumor				
Left	118	61	8	187 (54.20)
Right	98	55	5	158 (45.80)
Distal	195	13	1	209 (60.58)
Proximal	21	103	12	136 (39.42)
No. Enneking stage				
II	189	103	10	302 (87.54)
III	27	13	3	43 (12.46)
No. First operation method				
Amputation	67	38	5	110 (31.88)
Limb salvage	149	78	8	235(68.12)
No. Type of limb-salvage				
Expandable prosthesis	132	47	0	179 (76.17)
Modular prosthesis	17	31	0	48(20.43)
Tumor resection	0	0	8	8(3.40)
No. Follows-up				
Follow-up cases	198	112	13	323 (93.62)
Case of missing	18	4	0	22 (6.38)
No. Survival status				
Survive	100	61	6	167 (48.41)
Deaths	98	51	7	156 (45.22)
Follows-up time, M				
Mean	45.08	52.92	58.38	48.17
Median (range)	28 (1-191)	42.5 (4-184)	28 (3-243)	32 (1-243)

**Figure 2:**
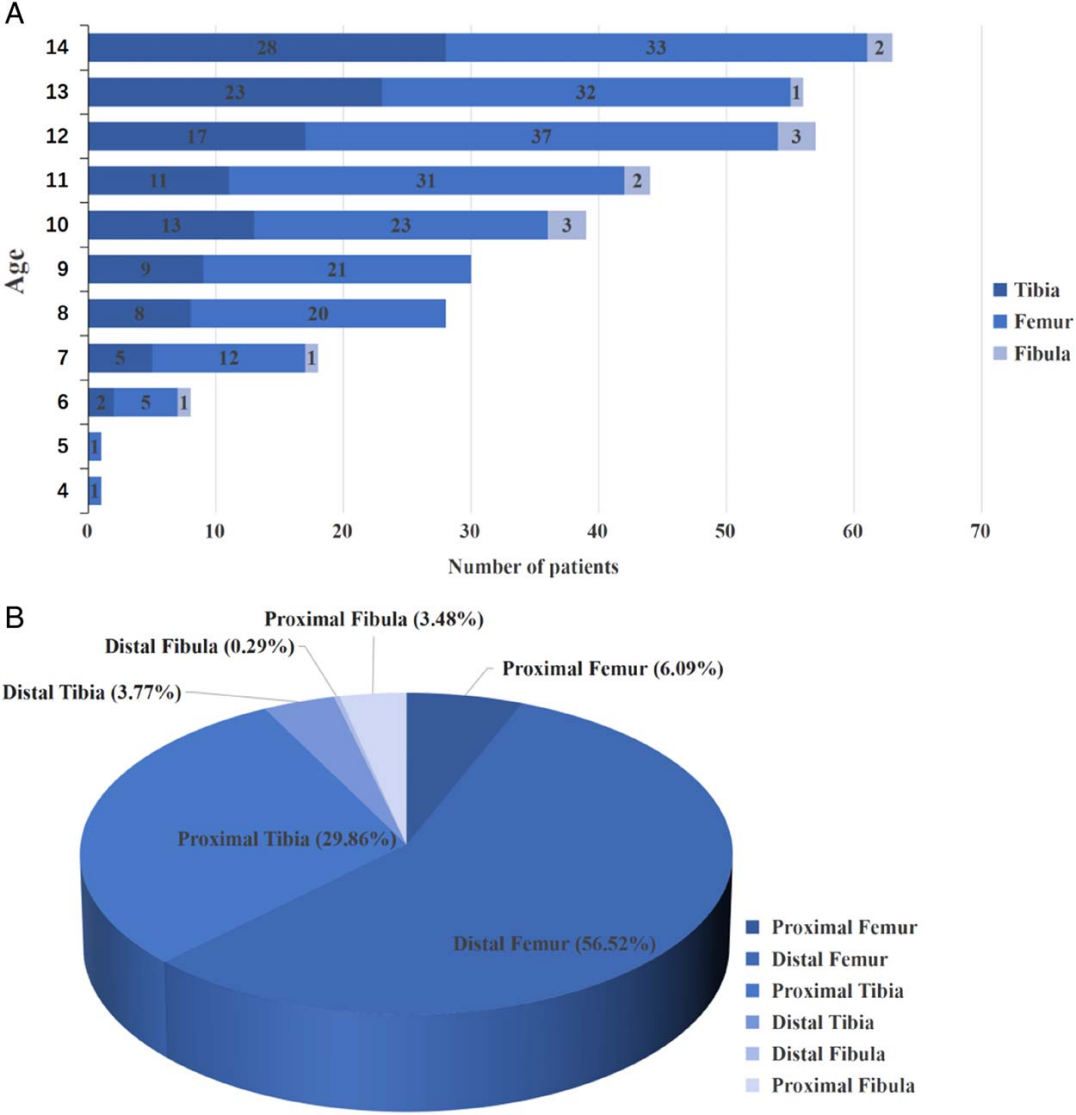
The distribution of age (A) and primary tumor site (B) of all patients.

**Figure 3:**
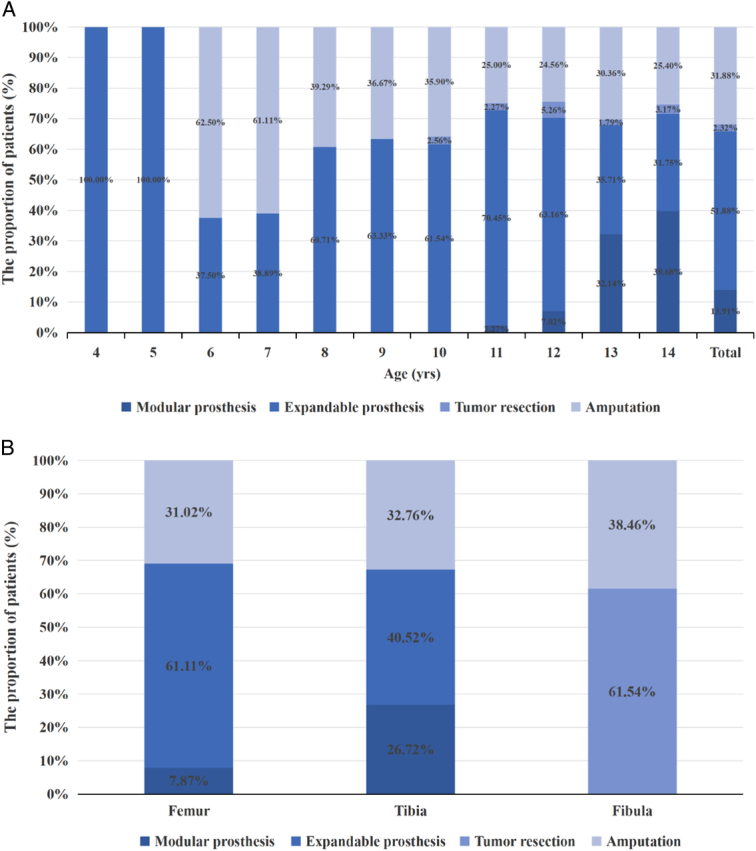
Surgical methods characteristics of age distribution (A) and surgical methods characteristics of tumor location (B).

The age of onset for pediatric patients was 10–14 years old (n=259, 75.07%) in our study. The proportion of low-age patients with femoral osteosarcoma (66.40%) was higher than that of patients with tibial osteosarcoma (29.60%). As individuals advanced in age, there was a gradual increase in the prevalence of tibial osteosarcoma among patients. Among high-age children, the prevalence of tibial osteosarcoma was observed to reach 35.91%, while the prevalence of femoral osteosarcoma reached 60.45% (Fig. 2A). Distal femur (56.52%) and proximal tibia (29.86%) were the predilection sites (Fig. 2B). The proportion of limb salvage operations increased with age, which was less than 40% when the patients were younger than 8 years old, about 60% for patients between 8 and 10 years old and increased to about 70% for patients older than 10 years old (Fig. 3A). The limb salvage rate of the first surgical treatment was similar for patients with tumor in femur (68.98%), tibia (67.24%), and fibula (61.54%). Pediatric patients with femoral osteosarcoma had applied more expandable prosthesis than those with tibial osteosarcoma (61.11% vs. 40.52%, *P*=0.001) (Fig. 3B). In the limb-salvage group, almost all of patients under 12 years old were applied expandable prosthesis. Modular prosthesis was more applicable for patients 12 years or older, especially for girls. Girls (34.18%, 27/79) over 12 years old had a higher modular prosthesis replacement rate than boys (20.62%, 20/97) (*P*=0.043). 48.98% (24/49) girls and 27.14% (19/70) boys at 13 and 14 years old applied modular prosthesis (*P*=0.015). Supplement Table 1, Supplemental Digital Content 2, http://links.lww.com/JS9/C152.

### Oncological outcomes

**Figure 4:**
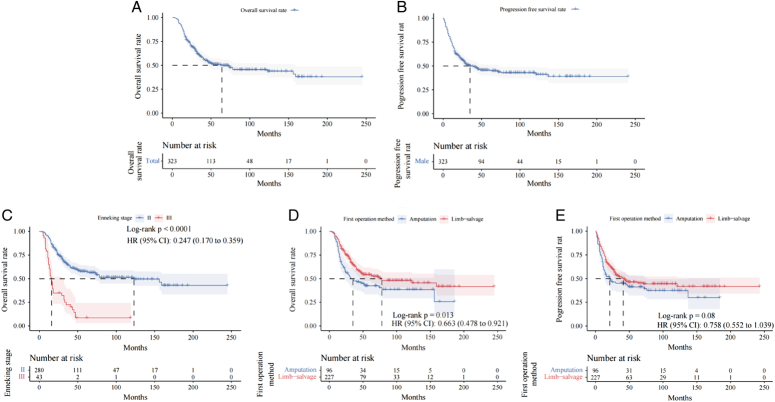
Kaplan–Meier curves showing OS and PFS in pediatric patients with significant difference oncological outcomes.

Until the last follow-up, 22 (6.3%) cases were lost to follow-up, 156 (45.22%) patients died and 167 (48.41%) patients survived (Table 1). Univariate analysis was conducted to examine prognostic factors for survival. The study revealed the OS was 50.3% at 5 years, 43.8% at 10 years, and 37.9% at 15 years. Additionally, the PFS was found to be 44.8% at 5 years and 41.1% at 10 years (Fig. 4A and B). The Enneking stage II [*P*<0.0001, HR (95% CI): 0.247 (0.170–0.359)] and Limb-salvage group [*P*=0.013, HR (95% CI): 0.663 (0.478–0.921)] presented better overall survival rate. The 5, 10, and 15 years overall survival rates for Enneking stage II were 57.1%, 49.6%, and 43.0%, respectively, which were 8.5%, 0%, and 0% for Enneking stage III. The 5, 10, and 15-year overall survival rates for patients with limb salvage were 53.4%, 45.6%, and 41.8% compared with 42.5%, 38.6%, 25.7% for patients with amputation (Fig. 4C and D). The progression-free survival rate displayed no significant difference between the Limb-salvage group and the amputation group [PFS, 5-year PFS 45.5% vs. 39.4%, 10-year PFS 41.8% vs. 29.9%, *P*=0.08, HR (95% CI): 0.758 (0.552–1.039)] (Fig. 4E). There were no statistically significant differences in sex, age, and tumor location in relation to OS and PFS (*P*>0.05). (Supplement Figure 1 A to F, Supplemental Digital Content 3, http://links.lww.com/JS9/C153).

The incidence of local recurrence rate (LRR) was 9.28% (32/345), and the metastatic rate was 28.99% (100/345) (Table 2). The median recurrence-free interval was 11 months（range 1 to 29 months, metastasis-free interval was 9 months(range 1–150). There were no significant differences in LRR among the tumor location, age, or Enneking stage (*P*>0.05). The LRR in limb salvage (29/235, 12.34%) was significantly higher than that of amputation (3/110, 2.73%) (log-rank *χ*^2^=8.514, *P*=0.004). Long recurrence-free intervals were observed in the limb-salvage group, Enneking stage II, and femoral osteosarcoma patients. The limb-salvage group (*P*=0.006) and Enneking stage II (*P*=0.0001) patients had longer relapse intervals than the amputation group and Enneking stage III. As for the tumor location, the patients with tumor in the femur had longer relapse-free intervals than those with tumor in the fibula (13 mo vs. 2.5 mo, *P*=0.025) (Table 2). There were no statistically significant differences among patients with different surgical methods, tumor location, and the age in the metastasis rate and the time of metastasis-free interval(*P*>0.05). (Table 2).

**Table 2:** The relapse and metastasis rate of different independent group.

	Relapse, No. (%)			Metastasis, No. (%)		
	YES	NO	Overall recurrence-free interval M, median (range)	YES	NO	Overall metastasis-free interval M, median (range)
Surgical method						
Amputation	3 (2.73)	107 (97.27)	8 (8)	31 (28.18)	79 (71.82)	6 (1–137)
Limb salvage	29 (12.34%)^a^	206 (87.66)	13 (1–29)^a^	69 (29.36)	166(70.64)	11.5 (1–150)
Tumor location						
Femur	21 (9.72)	195 (90.28)	13 (3–29)^c^	65 (30.09)	151 (69.91)	8 (1–137)
Tibia	9 (7.76)	107 (92.24)	7 (1–29)	33 (28.45)	83 (71.55)	12 (1–150)
Fibula	2 (15.38)	11 (84.62)	2.5 (2–3)	2 (15.38)	11 (84.62)	9.5 (7–12)
Age group	125	220				
Low age	9 (7.20)	116 (92.8)	8 (5–28)	32 (25.60)	93 (74.40)	7 (1–40)
High age	23 (10.45)	197 (89.55)	13 (1–29)	68 (30.91)	152 (69.09)	10 (1–150)
Enneking stage						
Enneking II	27 (8.94)	275 (91.06)	13 (4–29)	N/A	N/A	N/A
Enneking III	5 (11.63)	38 (88.37)	2 (1–6)^b^	N/A	N/A	N/A
Total	32 (9.28)	313 (90.72)	11 (1–29)	100 (28.99)	245 (71.01)	9 (1–150)

a *P*<0.05, compare with amputation.

b *P*<0.05, compare with Enneking II.

c *P*<0.05, compare with fibula.

N/A, not applicable.

### Complications

Thirteen patients with fibula osteosarcoma were included in the study. Eight of them underwent tumor resection without endoprosthesis replacement and were not included in the complication analysis. Amputation was performed on five additional patients, and only two of these patients experienced complications (one case of soft-tissue failure and two cases of tumor progression).

**Figure 5:**
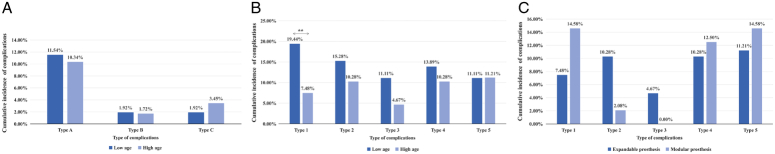
The characteristics of complications (A) by amputation and (B) by expandable prosthesis replacement of different ages, (C) by different type of limb salvage in high-age.

In general, there were 136 complications (40.96%) in 332 patients. Among these, 88 complications (88/216, 40.74%) occurred in femoral osteosarcoma, and 48 complications (48/116, 41.38%) occurred in tibial osteosarcoma (Table 3). There was no statistically significant difference in the number of complications between different tumor locations. Complications in the limb salvage group (119/227, 52.42%) were higher than that of the amputation group (17/105, 16.19%) (*P*<0.05). Specifically, the main complications of the limb-salvage group were soft tissue failure (29/227, 12.78%), periprosthetic infection (27/227, 11.89%), tumor progression (27/227, 11.89%), aseptic loosening (23/227, 10.13%) and structural failure (13/227, 5.73%). The main complications were soft-tissue failure (12/105, 11.43%), tumor progression (3/105, 2.86%), and infection (2/105, 1.90%) in the amputation group (Table 3). The incidence of complications varied among different tumor locations. The patients with osteosarcoma in the tibia suffered more prosthesis-related complications, including soft tissue failure (12/78, 15.38%) and periprosthetic infections (11/78, 14.10%), which was mostly owing to the poor soft tissue cover in the proximal tibia. While the most common prosthesis-related complication were aseptic loosening (18/149, 12.08%) and soft tissue failure (17/149, 11.41%) in patients with femoral osteosarcoma (Table 3). Pediatric patients who received amputation had the most complications with soft-tissue failure in both the low-age (11.54%) and high-age group (10.34%), but all types of complication showed no statistical difference between age groups (*P*>0.05) (Fig. 5A).

**Table 3:** Frequency of different types of complications.

	Tumor location			Prosthesis type	
Complication (No. (%))	Femur	Tibia	Total	Expandable prosthesis	Modular prosthesis
Limb salvage					
Total	78 (52.35%)^b^	41 (52.56%)^b^	119 (52.42%)^b^	98 (54.75%)	21 (43.75%)
Soft-tissue failure	17 (11.41%)	12 (15.38%)	29 (12.78%)	22(12.29%)	7(14.58%)
Aseptic loosening	18 (12.08%)	5 (6.41%)	23 (10.13%)	22(12.29%)^a^	1(2.08%)
Structural failure	8 (5.37%)	5 (6.41%)	13 (5.73%)	13(7.26%)^a^	0
Periprosthetic infection	16 (10.74%)	11 (14.10%)	27 (11.89%)	21 (11.73%)	6 (12.5%)
Tumor progression	19 (12.75%)	8 (10.26%)	27 (11.89%)	20 (11.17%)	7 (14.58%)
Amputation					
Total	10 (14.93%)	7 (18.42%)	17 (16.19%)	N/A	N/A
Soft-tissue failure	7 (10.45%)	5 (13.16%)	12 (11.43%)	N/A	N/A
Infection	1 (1.49%)	1 (2.63%)	2 (1.90%)	N/A	N/A
Tumor progression	2 (2.99%)	1 (2.63%)	3 (2.86%)	N/A	N/A
Total	88 (40.74%)	48 (41.38%)	136 (40.96%)	N/A	N/A

a *P* less than 0.05, compare with modular prosthesis.

b *P* less than 0.05, compare with amputation.

In the limb-salvage group, there were 98 cases of complications (98/179, 54.75%) in the expandable prosthesis group and 21 cases of complications (21/48, 43.75%) in the modular prosthesis group, but no statistically significant difference was observed (*P*=0.175). Both groups had a high risk of soft tissue failure, periprosthetic infection, and tumor progression. The expandable prosthesis group had a higher risk of aseptic loosening (12.29% vs. 2.08%, *P*<0.05) and structure failure (7.26% vs. 0%, *P*<0.05) than the modular prosthesis group (Table 3). The Low-age group had a higher frequency of complications than high-age group (68.06% vs. 34.08%, *P*=0.001), especially for prosthesis-related complications (59.72% vs. 27.37%, *P*=0.001), which included soft tissue failure (19.44% vs. 8.38%, *P*=0.040), aseptic loosening (15.28% vs. 6.70%, *P*=0.080), structure failure (11.11% vs. 3.35%, *P*=0.017), and periprosthetic infection (13.89% vs. 9.50%, *P*=0.804) (Supplement Table 2, Supplemental Digital Content 2, http://links.lww.com/JS9/C152).

In the expandable prosthesis group, the interval between operation and aseptic loosening ranged from 3.37 to 107.27 months, with an average of 28.16 months. The group experienced 10 cases of periprosthetic fracture and 3 cases of implant fracture, but none of them experienced structure failure again until the last follow-up (Supplement Table 3, Supplemental Digital Content 2, http://links.lww.com/JS9/C152). In the expandable prosthesis group, Low-age patients had more prosthesis-related complications than High-age patients in all four types, especially the proportion of soft-tissue failure in low-age patients was higher than in high-age patients (19.44% vs. 7.48%, *P*=0.017) (Fig. 5B). The cumulative incidence of complications in high-age group with limb salvage was further analyzed, soft-tissue failure (14.58%) was the most common prosthesis-related complication in patients underwent modular prosthesis. Aseptic loosening (10.28%) and periprosthetic infection (10.28%) were the most common type in patients who underwent expandable prosthesis, but all types of complications showed no statistically significant differences (*P*>0.05) (Fig. 5C).

### Complication related secondary amputation

**Figure 6:**
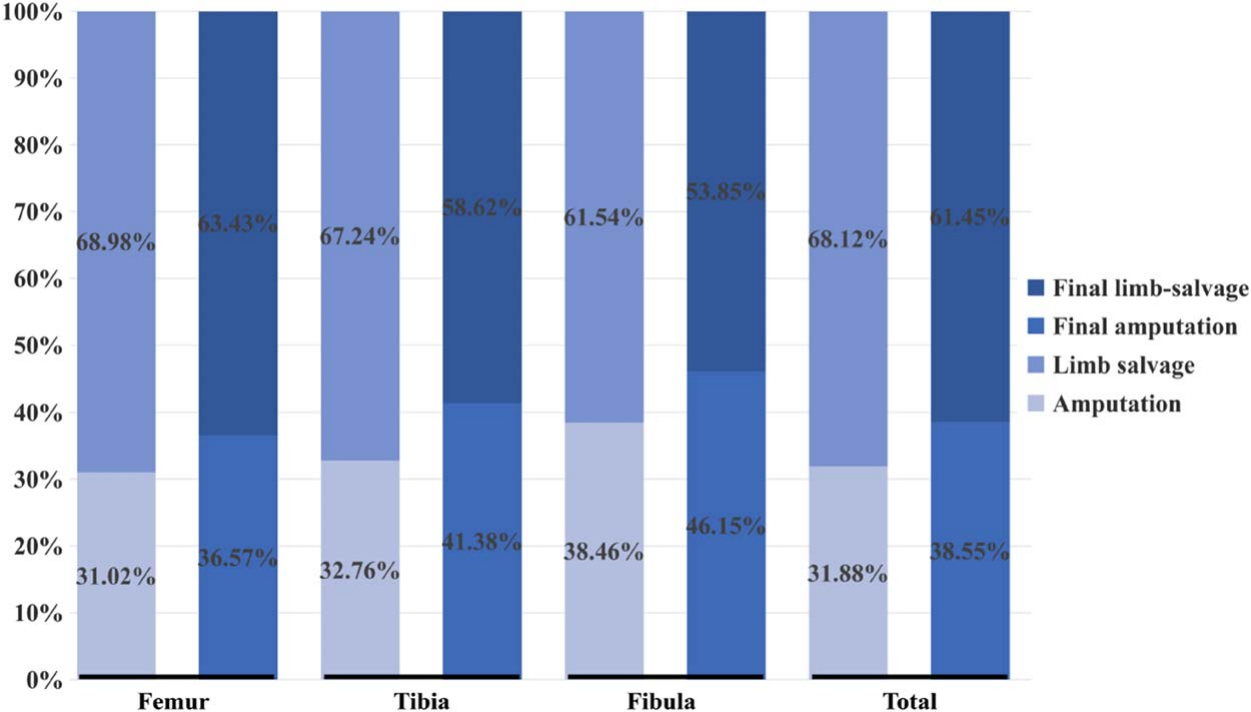
Characteristics of patients’ final surgical methods.

A total of 14 cases (14/235, 5.96%) of secondary amputation due to recurrence after the first limb salvage surgery treatment, and 9 cases (9/235, 3.83%) of amputation due to repeated infection around the surgical site (Supplement Table 4, Supplemental Digital Content 2, http://links.lww.com/JS9/C152). Finally, there were 212 patients (212/345, 61.45%) with limb-salvage and 133 patients (133/345, 38.55%) with amputation (Fig. 6). Among those patients who underwent surgery, low-age patients had a higher proportion of amputation rate compared with high-age patients (first amputation rate 41.60% vs. 26.36%, *P*=0.005. final amputation rate 48.00% vs. 33.18%, *P*=0.009). However, the reasons for secondary amputation showed no differences (*P*>0.05). The detailed records of secondary amputation are shown in Supplement Table 4, Supplemental Digital Content 2, http://links.lww.com/JS9/C152.

### Prosthesis survival

**Figure 7:**
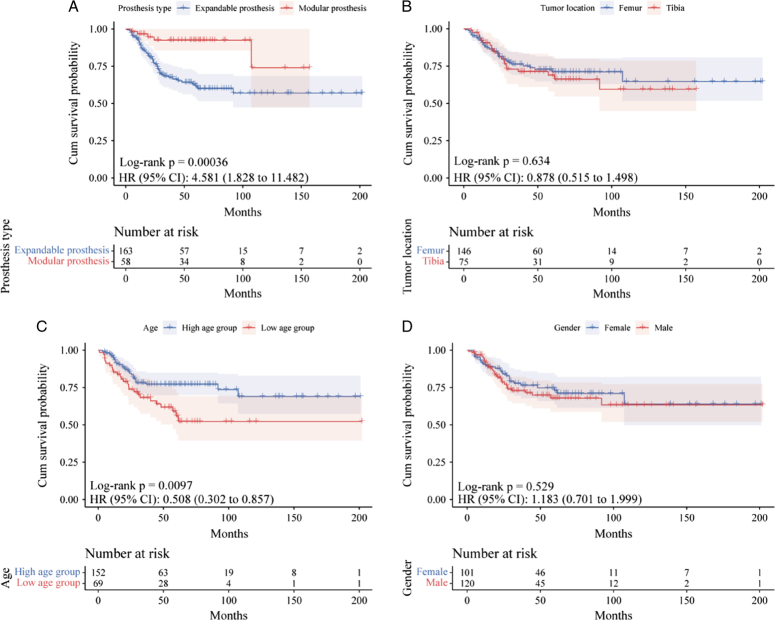
Kaplan–Meier prosthesis survival curves for patients with different independent group.

Using revision or removal of the stemmed components for any reason as an endpoint. Subgroup analysis for the prosthesis survival rate was made based on the type of prosthesis, tumor location, age, and gender. The expandable prosthesis group had a lower prosthesis survival rate than the modular prosthesis group [*P*=0.00036, HR (95% CI): 4.581 (1.828–11.482)], the 5-year prosthesis retention survival rate was 61.4% in the expandable prosthesis group and 83.3% in the modular prosthesis group, the 10-year prosthesis survival rate was 56.9% in the expandable prosthesis group and 74.0% in the modular prosthesis group (Fig. 7A). Tumor location was not associated with prosthesis survival [*P*=0.634, HR (95% CI): 0.878 (0.515–1.498)] (Fig. 7B).

The age of the patient was a strong determinant of prosthesis survival, the high-age group showed higher prosthesis survival than the low-age group [*P*=0.0097, HR (95% CI): 0.508 (0.302–0.857)], the prosthesis survival rate was 85.3% in the low-age group and 94.6% in the high-age group at 1-year, 67.2% in the low-age group and 77.1% in the high-age group at 3-year, 52.2% in the low-age group and 75.4% in the high-age group at 5-year (Fig. 7C). The gender was not associated with prosthesis survival [*P*=0.529, HR (95% CI): 1.183 (0.701–1.999)] (Fig. 7D).

### MSTS score of patients after surgery

The mean follow-up was 48.17 months (range 1–243 mo). A total of 22 patients did not have functional outcomes recorded because they were lost to follow-up (Table 1). Ultimately, 323 patients were evaluated functionally using the MSTS scoring system for limb salvage or amputation, and subgroup analysis of the MSTS score was made based on the tumor location, surgical method, type of prosthesis, and age.

The MSTS score of different tumor locations with limb salvage showed no significant differences among the femur, tibia, and fibula (*P*>0.05). The Limb-salvage group had a higher MSTS score (21.83±3.84) compared with the amputation group (6.68±1.18). However, patients who received amputation had lower scores in all items except for pain (*P*<0.05) (Table 4). Furthermore, patients who received modular prosthesis had a higher global score of 23.19±3.87 compared with those with expendable prosthesis 21.35±3.59 (*P*<0.05). (Table 5). Low-age group patients only underwent expandable prosthesis or amputation, and there was a significant difference between them (*P*<0.05). In the High-age group, Modular prosthesis (23.50±3.36) showed a better global score than expandable prosthesis (21.37±3.62) (*P*<0.05) (Table 4).

**Table 4:** The MSTS score of patients in different age group.

Variable mean±SD	Pain	Function	Emotional acceptance	Use of supports	Walking ability	Gait	Global
Low age							
Expandable prosthesis	4.06±0.77	3.53±0.91^b^	3.65±1.03^b^	3.65±0.79^b^	3.59±0.76^b^	3.25±0.82^b^	21.72±3.60^b^
Amputation	4.11±0.73	0.91±1.00	1.17±1.01	0.32±1.07	1.19±0.88	0.30±1.00	8.00±4.71
High age							
Expandable prosthesis	3.91±0.74^a^	3.58±0.91^b^	3.44±1.19^a^ ^b^	3.63±0.82^b^	3.52±0.75^b^	3.29±0.76^b^	21.37±3.62^a^ ^b^
Modular prosthesis	4.50±0.58	3.75±0.70^b^	4.04±0.92^b^	3.94±0.93^b^	3.73±0.74^b^	3.54±0.90^b^	23.50±3.36^b^
Amputation	4.08±0.58	0.75±0.44	0.92±0.68	0.00±0.00	1.02±0.48	0.00±0.00	6.77±1.31

a *P* less than 0.05, compare with modular prosthesis in high age group.

b *P* less than 0.05, compare with amputation of corresponding group.

**Table 5:** The MSTS score of patients in different independent group.

Variable mean±SD	Pain	Function	Emotional acceptance	Use of supports	Walking ability	Gait	Global
Tumor location							
Femur	3.94±0.67	3.69±0.92	3.49±1.18	3.61±0.75	3.61±0.74	3.41±0.74	21.75±3.51
Tibia	4.34±0.82	3.39±0.85	3.86±0.91	3.79±1.07	3.49±0.87	3.14±1.00	22.01±4.12
Fibula	4.13±1.36	3.13±0.99	3.88±0.99	3.75±1.16	3.5±1.41	3.00±1.3	21.38±6.59
Surgical method							
Limb salvage	4.08±0.77	3.57±0.91^b^	3.63±1.10^b^	3.68±0.89^b^	3.56±0.81^b^	3.30±0.81^b^	21.83±3.84^b^
Amputation	4.06±0.66	0.71±0.52	0.92±0.59	0.00±0.00	0.99±0.42	0.00±0.00	6.68±1.18
Prosthetic type							
Expandable prosthesis	3.97±0.75^a^	3.57±0.90	3.52±1.15^a^	3.65±0.80	3.57±0.75	3.28±0.78	21.57±3.59^a^
Modular prosthesis	4.50±0.58	3.69±0.88	4.04±0.92	3.83±1.08	3.65±0.91	3.48±1.03	23.19±3.87

a *P* less than 0.05, compare with modular prosthesis.

b *P* less than 0.05, compare with amputation.

## Discussion

Treatment of osteosarcoma in children’s lower limbs presents unique challenges due to the immature bone and soft tissue conditions, as well as the need to consider future growth potential. There is a lack of studies with long-term outcomes in pediatric lower-limb osteosarcoma cases. This retrospective study of pediatric patients under 14 years old represents the largest single-center analysis of its kind. This study provides valuable insights into the oncological and functional outcomes of lower-limb osteosarcoma in children.

The prognosis of osteosarcoma has witnessed substantial enhancements as a result of technological advancements, breakthroughs in chemotherapy, and the emergence of diagnostic imaging techniques. In the present study, the overall survival rates at 5, 10, and 15 years were determined to be 50.3%, 43.8%, and 37.9%, respectively. Notably, the overall survival rate for Enneking stage II reached 57.1% at 5 years, 49.6% at 10 years, and 43.0% at 15 years. There is a consensus that patient age poses a significant challenge for oncologists. Our study revealed that children who received chemotherapy and surgical resection treatment had a higher overall survival rate compared with adults^12–14^. Patients with limb-salvage surgery had a higher 5-year OS (53.4% vs. 42.5%) than the amputation group which was consistent with the finding of Li *et al*.^15^, and poorer oncological outcomes in pediatric patients were not explained by gender, age, and tumor location at presentation.

Local relapse rate (LRR) is an important predictor for evaluating the efficacy of osteosarcoma outcomes. LRR is expected to be higher in the limb-salvage group as a general rule as the margins in an amputation will usually be radical. The overall LRR was 9.28%, and the LRR in the limb-salvage group (12.34%) was significantly higher than that of the amputation group (2.73%). Other long-term follow-up studies have reported local relapse rates between 3% and 10% in limb-salvage^16–19^. Local recurrence in osteosarcoma is directly associated with the margins of excision and with the responsiveness of the primary tumor to chemotherapy^5^. The high LRR in this study may be attributed to the fact that all patients included were under 14 years old, patients and their families had a stronger desire to preserve their limbs even the crucial vessels were affected by the tumor, just as we reported before^20^. Alongside this inclination for limb salvage, the trend in tumor metastasis served as a crucial indicator for assessing the malignancy of the tumor during early diagnosis and treatment. Further analysis of 32 recurrent patients revealed that 25 out of 32 patients (78.13%) experienced lung metastasis. Among them, 5 cases had preoperative pulmonary metastasis and 20 cases had postoperative pulmonary metastasis. Long recurrence-free intervals were observed in the limb-salvage group, Enneking Stage II, and femoral osteosarcoma patients. In addition, metastasis and metastasis-free intervals did not exhibit significant differences in each subgroup.

The decision between amputation and limb salvage remains a controversial subject, particularly in young children. Worch *et al*.^21^ reported that very young patients (55.2%) had a higher proportion of amputation compared with older patients (27.3%). Liamb-sparing procedures pose a significant treatment challenge in pediatric patients, carrying a high risk of local tumor control and restoring postoperative joint function^22,23^. In this study, it was observed that the efficacy of conserving limbs through surgical intervention exhibited a positive correlation with age. Notably, patients below the age of 8 demonstrated a success rate of less than 40%, whereas those falling within the age range of 8 to 10 exhibited a success rate of approximately 60%. Encouragingly, patients aged 10 and above displayed a success rate of approximately 70%. Low-age pediatric patients had significantly higher amputation rates during the first surgery (41.60% vs. 26.36%) and final surgery (48.00% vs. 33.18%). The decision to proceed with amputation was solely made in situations where it was deemed unfeasible to attain effective tumor control or salvage a functional limb. Amputation for adult osteosarcoma is associated with older age, advanced stage, larger tumors, greater comorbidities, and lower income in National Cancer Database (NCDB)^24^. Remarkably, children have limited of effective communication and expression, which may lead to delays in diagnosis and surgical treatment. The discrepancy in limb length after limb salvage was more difficult to solve even with expandable prothesis in low-age patients, which may affect the function after bone maturation. Moreover all kinds of prosthesis related complications occurred more commonly in less than or equal to 10 yrs pediatric patients than that in less than 10 yrs pediatric patients as observed in our study. Younger patients present with suboptimal soft tissue conditions in their extremities, coupled with greater growth potential, leading to heightened complications such as soft-tissue failure, aseptic loosening, and leg length discrepancy (LLD). All kinds of prosthesis-related complications occurred more commonly in less than or equal to 10 yrs pediatric patients than in greater than 10 yrs pediatric patients as observed in our study. As a result, this requires multiple revision surgeries, thus amplifying the financial strain on patients and their families. We suggest that for pediatric patients under the age of 8 diagnosed with lower limb osteosarcoma, in cases where oncological factors permit limb preservation and options for artificial implants are limited due to the patient’s young age, biological reconstruction methods such as bone allograft^25–28^ and vascularized fiber graft (VFG)^27,29,30^ may improve functional outcomes and a reduced necessity for multiple revision surgeries. Tissue tolerance in pediatric patients increases after the age of 8, thereby making limb salvage surgery using extendable prostheses a viable option. This approach can yield favorable functional and aesthetic outcomes with an acceptable risk of complications.

Children had all of the complications found in adults treated with endoprosthesis expect pediatric-specific complications^31^. Henderson *et al*.^11^ reported that periprosthetic infection was the most common mode and soft-tissue failure was the least common failure for adults. However, soft-tissue failure was the most common mode of failure, and structural failure was the least common failure in the present pediatric patient series. The low-age group had a higher incidence of complications related to prostheses compared with the high-age group. Soft-tissue failure was the most frequent type of failure in the low-age group, while the high-age group tended to experience periprosthetic infection, which was similar to adults. Our findings align with previous research, affirming that the main complication in skeletally immature individuals is predominantly associated with soft tissue^32–34^, which may be owing to inadequate soft tissue storage in low-age patients. So, gentle surgical manipulation and reduced application of electrotome are necessary to decrease the surgical trauma and protect as much soft tissue as possible. If inadequate soft tissue to cover the prosthesis, transfer of nearby muscle flaps, such as gastrocnemius muscle flap or posterior thigh muscle translocation may be needed. In this study, aseptic loosening was the most common type of failure in expandable prosthesis, which was similar to the literature, as most of the authors reported it as the common mode of failure of expandable prosthesis^35–37^. Increased aseptic loosening of the prosthesis in pediatric patients may be owing to circumferential bone growth, which might widen the bone and the intramedullary canal, especially in younger pediatric patients. Hydroxyapatite coated collar or three-dimensional printed collar to increase bone ingrowth at the interface of prosthesis and bone may reduce the risk of aseptic loosening and sinking of prosthesis. Additionally, the effect of whether the angle of implant placement during surgery is perfectly aligned with the bone axis, and prosthesis implantation on periosteal blood supply are significant factors influencing the stability of prosthesis.

The choice of the definitive surgical procedure should consider the patient’s age and growth potential, the primary tumor’s location and its relationship to nearby structures, and the Enneking stage. Despite high complication rates in pediatric patients with lower-limb osteosarcoma, favorable functional outcomes and quality of life are observed after limb-salvage surgery. When the patient’s growth potential is less than 2 cm, it may be worth considering the use of modular prostheses, which can provide greater benefits. Targeted positive choices could be made to reduce the incidence of complications in patients with different risk factors.

In general, we summarized the characteristics of long-term oncological and functional outcomes in pediatric patients with lower-limb osteosarcoma, which was the largest single-center retrospective study. Our study increased the generalizability of our findings and facilitated evidence-based decision-making for the treatment of malignant pediatric lower-extremity osteosarcoma. Meanwhile, our work had certain limitations. Our primary focus was on amputation and internal prosthesis replacement in pediatric patients, but we lacked research on allograft, sterilization and reimplantation of bone, vascularized fibula graft, vascularized graft combined with allograft, bone distraction, and rotationplasty. Differences between age subgroups and longer-term follow-up and outcome evaluation warrant further study.

## Conclusions

The success rate of limb salvage in pediatric patients increases with age, and age should be considered when selecting the appropriate prosthesis. Pediatric patients with lower-limb osteosarcoma who underwent endoprosthesis replacement have a positive long-term outlook in terms of patient prognosis and manageable complications. For growing children who meet the limb salvage criteria for lower-limb osteosarcoma, endoprosthesis replacement is a valuable reconstruction option to preserve a functional limb, particularly for younger pediatric patients.

## Ethical approval

This study was approved by the Ethical Committee of SYSU First Affiliated Hospital. Application ID:[2023]331.

## Consent

All subjects gave written informed consent.

## Sources of funding

National Natural Science Foundation of China (NSFC), Grant/Award Number: 82273413.

National Natural Science Foundation of China (NSFC), Grant/Award Number: 81972507.

National Natural Science Foundation of China(NSFC), Grant/Award Number: 82103078.

Science and Technology Planning Project of Guangdong Province, China, Grant/Award Number:2023A1515010154.

Science and Technology Planning Project of Guangzhou, China, Grant/Award Number:202201010904.

## Author contribution

M.X., J.T., J.T., J.S., and C.Z. contributed to the conception of the study; M.X., J.T., Y.H., and C.Z. contributed significantly to analysis and manuscript preparation; M.X. performed the data analyses and wrote the manuscript; B.W., Z.Z., T.L., G.H., J.Y., X.X., J.S., and C.Z. helped perform the analysis with constructive discussions.

## Conflicts of interest disclosure

The authors declare no conflicts of interest.

## Research registration unique identifying number (UIN)

This study is a retrospective analysis of 345 pediatric patients with lower-limb osteosarcoma admitted to our department between 2000 and 2019.

Registration ID:NCT06162962.

Hyperlink:https://clinicaltrials.gov/study/NCT06162962.

## Guarantor

Changye Zou, zouchy@mail.sysu.edu.cn.

Jingnan Shen, shenjn@mail.sysu.edu.cn.

## Data availability statement

All data included in this study are available upon request by contact with the corresponding author.

Changye Zou, zouchy@mail.sysu.edu.cn.

## Provenance and peer review

None.

## Supplementary Material

SUPPLEMENTARY MATERIAL
