# Supplementary material for: Comparison of oncological and functional outcomes in Lower-limb osteosarcoma pediatric patients: a large single-center retrospective cohort study
Source: Int J Surg. 2024 Mar 12;110(7):4208–20. doi: 10.1097/JS9.0000000000001340 (PMC11254188; doi:10.1097/JS9.0000000000001340)
Supplement: SUPPLEMENTARY MATERIAL [file js9-110-4208-s002.docx]

**Supplement Table:**

**Supplement Table1. The characteristics of different type of limb-salvage in different age distribution.**

| Age,y | No. Expandable prosthesis (M/F) | No. Modular prosthesis (M/F) | No. Tumor resection (M/F) | No. Amputation (M/F) | No. Total (M/F) |
| --- | --- | --- | --- | --- | --- |
| 4 | 1(0/1) | 0 | 0 | 0 | 1(0/1) |
| 5 | 1(0/1) | 0 | 0 | 0 | 1(0/1) |
| 6 | 3(0/3) | 0 | 0 | 5(3/2) | 8(3/5) |
| 7 | 7(3/4) | 0 | 0 | 11(9/2) | 18(12/6) |
| 8 | 17(11/6) | 0 | 0 | 11(7/4) | 28(18/10) |
| 9 | 19(11/8) | 0 | 0 | 11(6/5) | 30(17/13) |
| 10 | 24(17/7) | 0 | 1(1/0) | 14(8/6) | 39(26/13) |
| 11 | 31(18/13) | 1(0/1) | 1(1/0) | 11(8/3) | 44(27/17) |
| 12 | 36(16/20) | 4(1/3) | 3(2/1) | 14(8/6) | 57(27/30) |
| 13 | 20(13/7) | 18(9/9) | 1(0/1) | 17(10/7) | 56(32/24) |
| 14 | 20(16/4) | 25(10/15) | 2(1/1) | 16(11/5) | 63(38/25) |
| Total | 179(105/74) | 48(20/28) | 8(5/3) | 110(70/40) | 345(200/145) |

**Note:**

M:Male, number of patients.

F:Female, number of patients.

**Supplement Table2. The postoperative complications’ characteristic in patients with prosthesis-replacement in different age.**

| **Age,y** | **Type 1**  **(No. [%])** | **Type 2**  **(No. [%])** | **Type 3**  **(No. [%])** | **Type 4**  **(No. [%])** | **Type 5**  **(No. [%])** | **Prosthesis related complications**  **(No. [%])** | **The number of patients with prosthesis related complications**  **(No. [%])** | **No. Total frequency of**  **complications (Type 1 to 5)** | **Total number of patients with complications (Type 1 to 5)(No. [%])** | **No. Total patient number** |
| --- | --- | --- | --- | --- | --- | --- | --- | --- | --- | --- |
| 4 | 0 | 1 (100%) | 1 (100%) | 0 | 1 (100%) | 2 (200%) | 1 (100%) | 3 | 1 (100%) | 1 |
| 5 | 0 | 0 | 0 | 0 | 1 (100%) | 0 | 0 | 1 | 1 (100%) | 1 |
| 6 | 0 | 1 (33.33%) | 0 | 0 | 0 | 1 (33.33%) | 1 (33.33%) | 1 | 1 (33.33%) | 3 |
| 7 | 1 (14.29%) | 0 | 1 (14.29%) | 2 (28.57%) | 1 (14.29%) | 4 (57.14%) | 7 (100 %) | 5 | 7 (100%) | 7 |
| 8 | 5 (29.41%) | 2 (11.76%) | 1 (5.88%) | 2 (11.76%) | 2 (11.76%) | 10 (58.82%) | 11(64.71%) | 12 | 12 (70.59%) | 17 |
| 9 | 4 (21.05%) | 2 (10.53%) | 3(15.79%) | 3(15.79%) | 3 (15.79%) | 12 (63.16%) | 12 (63.16%) | 15 | 15(78.95%) | 19 |
| 10 | 4 (16.67%) | 5 (20.83%) | 2 (8.33%) | 3 (12.50%) | 0 | 14 (58.33%) | 12 (50.00%) | 14 | 12 (50.00%) | 24 |
| **≤10** | 14 (19.44%)**^a^** | 11 (15.28%) | 8 (11.11%)**^a^** | 10 (13.89%) | 8 (11.11%) | 43 (59.72%)**^a^** | 44 (61.11%) **^a^** | 51 | 49 (68.06%) **^a^** | 72 |
| 11 | 3 (9.38%) | 3 (9.38%) | 3 (9.38%) | 3 (9.38%) | 4 (12.50%) | 12 (37.50%) | 11 (34.38%) | 16 | 15 (46.88%) | 32 |
| 12 | 4 (10.00%) | 4 (10.00%) | 1 (2.50%) | 6 (15.00%) | 5 (12.5%) | 15 (37.50%) | 14 (35.00%) | 20 | 19 (47.50%) | 40 |
| 13 | 3 (7.89%) | 2 (5.26%) | 0 | 4 (10.53%) | 4 (10.53%) | 9 (23.68%) | 8 (21.05%) | 13 | 12 (31.58%) | 38 |
| 14 | 5 (11.11%) | 3 (6.67%) | 1 (2.22%) | 4 (8.89%) | 6 (13.33%) | 13 (28.89%) | 12 (26.67%) | 19 | 15 (33.33%) | 45 |
| **>10** | 15 (8.38%) | 12 (6.70%) | 5 (3.35%) | 17 (9.50%) | 19 (10.61%) | 49 (27.37%) | 45 (25.14%) | 68 | 61 (34.08%) | 155 |
| **Total** | 29 (12.78%) | 23 (10.13%) | 13 (5.73%) | 27 (11.89%) | 27 (11.89%) | 92 (40.53%) | 89 (39.21%) | 119 | 110 (48.46%) | 227 |

**Note:**

Supplement table 2 included all limb-salvage osteosarcoma patients with prosthesis replacement.

Prosthesis related complications (no. [%]): the frequency of prosthesis related complications (type 1 to 4) occurred in different age;

Type1:log-rank χ2=4.209, P=0.040.

Type2:log-rank χ2=3.066, P=0.080.

Type3:log-rank χ2=5.662, P=0.017.

Type4:log-rank χ2=0.400, P=0.527.

Type5:log-rank χ2=0.062, P=0.804.

Prosthesis related complications: log-rank χ2=23.140, P=0.001.

The number of patients with prosthesis related complications: log-rank χ2=27.481, P=0.001.

Total number of patients with complications: log-rank χ2=22.718, P=0.001.

a P<0.05, compare with High-age group (>10 years old).

**Supplement Table 3. The characteristics of postoperative complications in patients with different type of limb-salvage in different age.**

| Age, y | Expandable prosthesis | | | | | | Modular prosthesis | | | | | |
| --- | --- | --- | --- | --- | --- | --- | --- | --- | --- | --- | --- | --- |
|  | Type 1 | Type 2 | Type 3 | Type 4 | Type 5 | Total  No. (%) | Type 1 | Type 2 | Type 3 | Type 4 | Type 5 | Total  No. (%) |
| 4 | 0 | 1 | 1 | 0 | 1 | 3 (1.68%) | 0 | 0 | 0 | 0 | 0 | 0 |
| 5 | 0 | 0 | 0 | 0 | 1 | 1 (0.56%) | 0 | 0 | 0 | 0 | 0 | 0 |
| 6 | 0 | 1 | 0 | 0 | 0 | 1 (0.56%) | 0 | 0 | 0 | 0 | 0 | 0 |
| 7 | 1 | 0 | 1 | 2 | 1 | 5 (2.79%%) | 0 | 0 | 0 | 0 | 0 | 0 |
| 8 | 5 | 2 | 1 | 2 | 2 | 12 (6.70%) | 0 | 0 | 0 | 0 | 0 | 0 |
| 9 | 4 | 2 | 3 | 3 | 3 | 15 (8.38%) | 0 | 0 | 0 | 0 | 0 | 0 |
| 10 | 4 | 5 | 2 | 3 | 0 | 14 (7.82%) | 0 | 0 | 0 | 0 | 0 | 0 |
| 11 | 3 | 3 | 3 | 3 | 3 | 15 (8.38%) | 0 | 0 | 0 | 0 | 1 | 1 (2.08%) |
| 12 | 3 | 4 | 1 | 5 | 4 | 17 (9.50%) | 1 | 0 | 0 | 1 | 1 | 3 (6.25%) |
| 13 | 2 | 2 | 0 | 2 | 3 | 9 (5.03%) | 2 | 1 | 0 | 4 | 1 | 8 (16.67%) |
| 14 | 0 | 2 | 1 | 1 | 2 | 6 (3.35%) | 4 | 0 | 0 | 1 | 4 | 9 (18.75%) |
| Total | 22 (12.29%) | 22 (12.29%) | 13 (7.26%) | 21(11.73%) | 20 (11.17%) | 98(54.75%) | 7 (14.58%) | 1 (2.08%) | 0 | 6 (12.50%) | 7 (14.58%) | 21 (43.75%) |

**Supplement Table 4. The characteristics of surgical methods of patients**

| Group | **Tumor location** | | | Age | |  |
| --- | --- | --- | --- | --- | --- | --- |
| Methods | Femur, No. (%) | Tibia, No. (%) | Fibula, No. (%) | Low-age, No. (%) | High-age, No. (%) | Total, No. (%) |
| Amputation | 67 (31.02%) | 38 (32.76%) | 5 (38.46%) | 52 (41.60%) ^b^ | 58 (26.36%) | 110 (31.88%) |
| Amputation for local recurrence | 9 (6.04%) | 4 (5.13%) | 1 (12.5%) | 5 (6.85%) | 9 (5.56%) | 14 (5.96%) |
| Amputation for repeated infection | 3 (2.01%) | 6 (7.69%) | 0 | 3 (4.11%) | 6 (3.70%) | 9 (3.83%) |
| Residual amputation | 79 (36.57%) | 48 (41.38%) | 6 (46.15%) | 60 (48.00%) ^b^ | 73 (33.18%) | 133 (38.55%) |
|  |  | | |  | |  |
| Limb salvage | 149 (68.98%) | 78 (67.24%) | 8 (61.54%) | 73 (58.40%) ^b^ | 162 (73.64%) | 235 (68.12%) |
| Residual limb-salvage | 137 (63.43%) | 68 (58.62%) | 7 (53.85%) | 65 (52.00%) ^b^ | 147 (66.82%) | 212 (61.45%) |
| Total | 216 (100.00%) | 116 (100.00%) | 13 (100.00%) | 125 (100.00%) | 220 (100.00%) | 345 (100.00%) |

**Note:**

First amputation: log-rank χ2=7.833, P=0.005. Final amputation: log-rank χ2=6.776, P=0.009.

b P<0.05, compare with High-age group (>10 years old).
