# Supplementary material for: Comparison of oncological and functional outcomes in Lower-limb osteosarcoma pediatric patients: a large single-center retrospective cohort study
Source: Int J Surg. 2024 Mar 12;110(7):4208–20. doi: 10.1097/JS9.0000000000001340 (PMC11254188; doi:10.1097/JS9.0000000000001340)

**Supplement Figure：**

**Supplement Figure 1. Kaplan-Meier curves showing OS and PFS in pediatric patients with no difference oncological outcomes.**


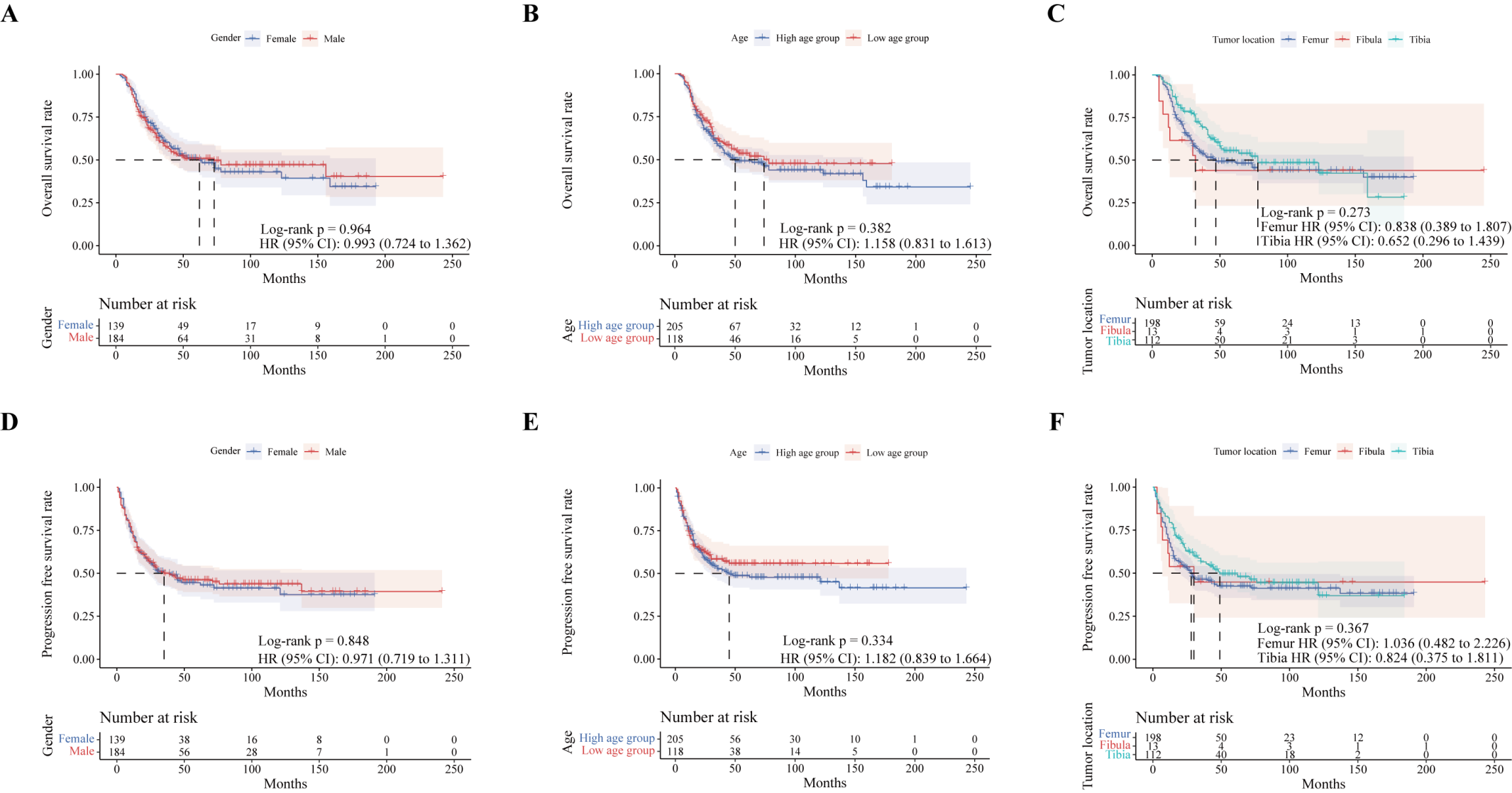

Supplement: SUPPLEMENTARY MATERIAL [file js9-110-4208-s003.docx]
